# Fifteenth Annual Meeting of the Miss. Valley Association of Dental Surgeons

**Published:** 1859-03

**Authors:** 


### FIFTEENTH ANNUAL MEETING OF THE MISS. VALLEY ASSOCIATION OF DENTAL SURGEONS.

### MINUTES.

The Association met according to adjournment, in the lecture room of the Ohio Dental College, Feb. 23, 1859.

Members present: James Taylor, Charles Bonsall, J. P. Ulrey, J. Taft, W. R. Webster, Joseph Richardson, H. A. Smith, Eli Collins, H. R. Smith, Geo. W. Keely, and Geo. Watt.

The President, W. R. Webster, in the chair. The minutes of the last meeting were read, and the roll of the members called.

Dr. C. Bonsall presented a paper from Dr. E. Osmond, resigning his membership; and as Dr. O. had paid to the Treasurer all dues to the association, his resignation was, on motion of Dr. Watt, unanimously accepted. The Treasurer, Dr. C. Bonsall, resported as follows:

Chas. Bonsall, Treasurer, in account with the M. V. A. of Dental Surgeons.

| | | |
|---|---|---|
| Dr. 1858, Feb. 17, To Balance in the Treasury................ | $120,00 |
| 1859, Feb. 23, To initiation fees and dues rec'd since | 53,00 |
| | $173,00 |
| Cr. 1859, Feb. 19, By Cash paid Janitor........................ | $ 5,00 |
| " " Dr. Watt for reporting.... | 15,00 |
| Both of the above by order of the Association. | |
| By balance in Treasurer's hands................................ | $153,00 |
| | $173,00 |

Drs. Ulrey and Richardson were appointed a committee to audit the above, who reported as follows:

The auditing committee having examined the Treasurer's account, find the same correct.

Joseph Richardson,<br>J. P. Ulrey, } Committee.

The Chair appointed Drs. Chas. Bonsall and H. A. Smith to fill the vacancies in the committee on membership, occasioned by the absence of Drs. E. Osmond and A. G. Stipher.

The Executive Committee presented the following report:

The Executive Committee would respectfully report the following programme for the order of business:

I. Reports of Officers.

II. Reports of Committees.

III. Reading of Essays.

IV. Election of Officers to take place at 2 o'clock P. M., Thursday.

V. Miscellaneous business, including the following subjects for discussion:

1. Treatment of Inflamed or sensitive denture.
2. Mechanical dentistry.
3. Treatment of dental periostitus and alveolar abscess.
4. To what extent are atmospheric plate's applicable to partial sets of teeth.
6. Anæsthetic agents, use of, in the extraction of teeth.
7. Filling Teeth.
8. Best means to secure a good denture.

J. P. Ulrey,<br>H. A. Smith,<br>C. N. Woodward, } Committee.

On motion, adjourned to meet at 2 o'clock, P. M.

FIRST DAY—AFTERNOON SESSION.

Met according to adjournment. Minutes read and approved.

The Committee on Irregularities being called on to report, the chairman stated that they had given the subject some attention, but were not prepared to report. Excused, by consent.

The Committee on Dental Progress obtained leave to report to-morrow, and also the Committee on the prize essay.

On motion of Dr. Watt, all members of the medical and dental professions present were invited to participate in the discussions.

Proceeded to the discussion of Miscellaneous subjects. No. 1 was taken up; and pending its discussion, adjourned to meet at 9, A. M., to-morrow.

SECOND DAY—MORNING SESSION.

Met according to adjournment. The President in the chair. Minutes of last session read and approved. No. 2 of Miscellaneous sujects was taken up.

On motion of Dr. Taft, the Constitution was amended, by a unanimous vote, so as to reduce the initiation fee to THREE DOLLARS and the annual dues to ONE DOLLAR.

The Committee on Dental Progress was instructed to report at 3, P. M., to-day.

The Committee on Membership presented the names of the following persons as candidates for admission to the Association:

Drs. S. F. Compton, Hillsborough, Ohio; Samuel Wardle, Cincinnati, Ohio; J. M. Lewis, Marion, Ill.; L. B. Hatch, Muncie, Ind.; B. B. Ward, Mobile, Ala.; C. H. James, Cincinnati, Ohio; S. B. Smith, Terre Haute, Ind.; J. B. Lindsey, Maysville, Ky.; all of whom were unanimously elected.

On motion of Dr. Taft, 3½, P. M., was set apart, for demonstrating the method of manipulations and "packing" the "coralite base" for artificial teeth.

On motion, adjourned to meet at 2 P. M.

SECOND DAY—AFTERNOON SESSION.

Met according to adjournment. Minutes read and adopted. Proceeded to the election of officers, which resulted as follows:

*President*, GEO. W. KEELY.
*Vice President*, H. R. SMITH.
*Corresponding Secretary*, JOSEPH RICHARDSON.

*Recording Secretary*, GEO. WATT.
*Treasurer*, CHAS. BONSALL.
*Executive Committee*, H. A. SMITH, E. COLLINS, L. B. HATCH.
*Committee on Membership*, S. WARDLE, J. P. ULREY, J. TAFT.
*Committee on Dental Progress*, H. A. SMITH, W. R. WEBSTER, GEO. WATT.
*Committee on Irregularity*, H. R. SMITH, J. TAYLOR, CHAS. BONSALL.

The Committee on Dental Progress presented their report which was read and accepted. (See page 310 of the present number of the *Register*.")

It being necessary for the President to leave the city, and the President elect being absent, the Vice President elect was called to the chair.

The method of "packing" the "coralite base" was thoroughly demonstrated by J. T. Toland and his assistants. This base is the vulcanized gutta percha. The thanks of the Association were, on motion, returned to Mr. Toland and assistants for their instruction in the above process.

On motion, adjourned till 9, A. M., to-morrow.

THIRD DAY—MORNING SESSION.

Minutes read and approved. Dr. Taft exhibited a practical case of "coralite base," which had been worn a few weeks with great satisfaction; a silver set having previously failed in the same mouth.

Mr. Toland exhibted the pieces which were "packed" in the presence of the Association yesterday, now vulcanized.

No. 3, of Miscellaneous subjects, was taken up; and, during its discussion, Dr. C. Bonsall, by consent, introduced an interesting case of irregularity which had been under treatment in the College Infirmary during the session. All seemed highly pleased with the appliances in use, and with the result of the treatment thus far.

By request, Mr. Toland described the various manipulations used in preparing work for vulcanizing.

Adjourned till 2, P. M.

THIRD DAY—AFTERNOON SESSION.

Met according to adjournment. G. W. Keely, the President elect, in the chair.

No. 5, Miscellaneous subjects, was discussed, and Nos. 6, 7 and 8 were postponed, No. 4 having been, by consent, considered in connection with No. 2.

On Motion of Dr. Bonsall, the Treasurer was instructed to pay to Geo. Watt $15 for reporting the discussions, and $8 to the janitor for his services during the meeting.

On motion, adjourned to meet in the Dental College, February 21st, 1860, at 10 o'clock, A. M.

